# Linoleic acid isomerase gene *FgLAI12* affects sensitivity to salicylic acid, mycelial growth and virulence of *Fusarium graminearum*

**DOI:** 10.1038/srep46129

**Published:** 2017-04-07

**Authors:** Ya-Zhou Zhang, Zhen-Zhen Wei, Cai-Hong Liu, Qing Chen, Bin-Jie Xu, Zhen-Ru Guo, Yong-Li Cao, Yan Wang, Ya-Nan Han, Chen Chen, Xiang Feng, Yuan-Yuan Qiao, Lu-Juan Zong, Ting Zheng, Mei Deng, Qian-Tao Jiang, Wei Li, You-Liang Zheng, Yu-Ming Wei, Peng-Fei Qi

**Affiliations:** 1 Triticeae Research Institute, Sichuan Agricultural University, Chengdu, Sichuan 611130, China; 2 Agronomy College, Sichuan Agricultural University, Chengdu, Sichuan 611130, China

## Abstract

*Fusarium graminearum* is the major causal agent of fusarium head blight in wheat, a serious disease worldwide. Linoleic acid isomerase (LAI) catalyses the transformation of linoleic acid (LA) to conjugated linoleic acid (CLA), which is beneficial for human health. We characterised a *cis*-12 *LAI* gene of *F. graminearum (FGSG_02668*; *FgLAI12*), which was downregulated by salicylic acid (SA), a plant defence hormone. Disruption of *FgLAI12* in *F. graminearum* resulted in decreased accumulation of *cis*-9,*trans*-11 CLA, enhanced sensitivity to SA, and increased accumulation of LA and SA in wheat spikes during infection. In addition, mycelial growth, accumulation of deoxynivalenol, and pathogenicity in wheat spikes were reduced. Re-introduction of a functional *FgLAI12* gene into Δ*FgLAI12* recovered the wild-type phenotype. Fluorescent microscopic analysis showed that FgLAI12 protein was usually expressed in the septa zone of conidia and the vacuole of hyphae, but was expressed in the cell membrane of hyphae in response to exogenous LA, which may be an element of LA metabolism during infection by *F. graminearum*. The *cis*-12 LAI enzyme encoded by *FgLAI12* is critical for fungal response to SA, mycelial growth and virulence in wheat. The gene *FgLAI12* is potentially valuable for biotechnological synthesis of *cis*-9,*trans*-11 CLA.

*Fusarium graminearum* (teleomorph *Gibberella zeae* [Schwein.] Petch) is an ascomycete fungus that causes fusarium head blight (FHB) in wheat. FHB can lead to yield loss and accumulation of trichothecene mycotoxins (predominantly deoxynivalenol [DON]) in wheat seeds, which threatens human and animal health^1^,^2^,^3^. Control of FHB by using natural resistance and traditional breeding in wheat is difficult, and thus FHB remains a serious disease worldwide^4^,^5^.

Linoleic acid (LA; *cis*-9,*cis*-12 C18:2) plays an important role in wheat resistance to *F. graminearum* infection. Accumulation of LA is greater in FHB-resistant wheat lines than in susceptible lines^6^, suggesting that LA may contribute to FHB resistance. Enhanced accumulation of LA and other free fatty acids can reinforce the cuticle, which acts as a barrier to pathogen entry^7^. LA is metabolised by linoleate diol synthase (LDS)^8^,^9^ and linoleic acid isomerase (LAI) in fungi (Fig. 1). LDS has been well studied in fungi, and is involved in fungal development, reproduction, synthesis of trichothecenes and cytochrome metabolism^8^,^9^,^12^,^13^. However, limited knowledge on LAI in fungi is available.

Activity of LAI was first analysed in the bacterium *Butyrivibrio fibrisolvens*^14^ and subsequently in the red alga *Ptilota filicina*^15^. To date, the enzyme has been detected mostly frequently in bacteria^16^,^17^. LAI catalyses the transformation of LA to conjugated linoleic acid (CLA) in microbes. Two categories of LAI (i.e., *cis*-9 and *cis*-12) have been identified: *cis*-9 LAI catalyses conversion of LA to *trans*-10,*cis*-12 CLA (*trans*-10,*cis*-12 C18:2); and *cis*-12 LAI transforms LA to *cis*-9,*trans*-11 CLA (*cis*-9,*trans*-11 C18:2). *Trans*-10,*cis*-12 CLA and *cis*-9,*trans*-11 CLA are then transformed into *trans*-10 C18:1 and *trans*-11 C18:1, respectively, by unknown enzymes (Fig. 1). In fungi, the gene encoding *cis*-9 LAI (*PAI*) was identified in *Propionibacterium acnes*^10^,^11^,^18^. Activity of *cis*-12 LAI was measured in *Clostridium sporogenes*; however, no *cis*-12 *LAI* gene has yet been reported^19^.

In this study, we characterised a *cis*-12 *LAI* gene of *F. graminearum (FGSG_02668*; *FgLAI12*; *cis*-12 LAI of *F. graminearum*), which was downregulated by salicylic acid (SA), a plant defence hormone^20^. The objectives were to investigate the function of *FgLAI12* in LA metabolism and to clarify the gene’s effects on fungal response to SA, fungal development, synthesis of DON and pathogenicity in wheat.

## Results

### Deletion and complementation of *FgLAI12*

The *FgLAI12* gene contains three exons and two introns (Fig. 2A). To determine the function of *FgLAI12* in *F. graminearum*, knockout mutants (Δ*FgLAI12*) and complementation mutants (C-*FgLAI12*) were created (Fig. 2A,B). The mutants were verified by PCR (Fig. 2C,D) and sequencing (data not shown).

The copy number of integrated T-DNA inserts was determined by targeting hygromycin B phosphotransferase genes (*HPH*). As expected, *HPH* was not detected in the wild type (WT). One and two normalised copies of *HPH* were detected in the Δ*FgLAI12* and C-*FgLAI12* mutants, respectively, confirming the single insertion of the T-DNA construct for gene knockout and complementation (Fig. 2E).

Expression of *FgLAI12* under treatment with 3 mmol L^−1^ SA or 10 mg mL^−1^ LA was determined by reverse transcription-PCR (RT-PCR). As expected, *FgLAI12* expression was downregulated by SA and upregulated by LA (Fig. 2F).

### *FgLAI12* catalyses conversion of LA to *cis*-9,*trans*-11 CLA

**Figure 1:**
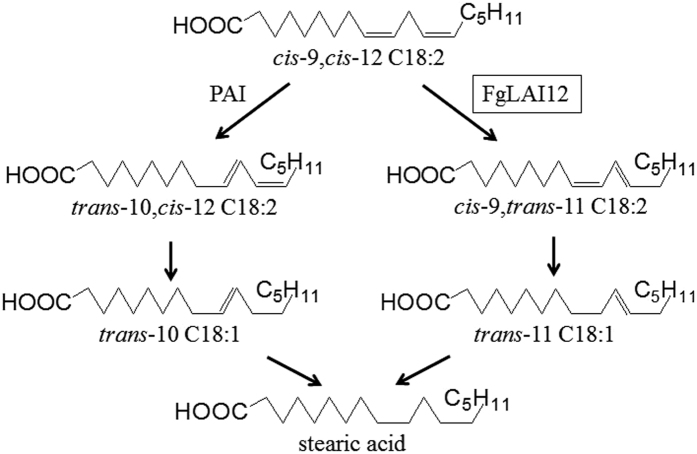
Schematic illustration of LA isomers. FgLAI12 is marked by a black box. PAI, *Propionibacterium acnes* cytosolic LA isomerase^10^,^11^.

Three peaks were detected in gas chromatography–mass spectrometry (GC-MS) analysis of compounds derived from LA (Fig. 3A), namely LA (the first peak), stearic acid (the second peak) and *cis*-9,*trans*-11 CLA (the third peak), by matching with GC-MS spectrometry gallery. The peaks for LA, stearic acid and *cis*-9,*trans*-11 CLA in GC-MS were confirmed by using of corresponding standard chemicals (Fig. 3B). In the presence of exogenous LA, enzyme extracts from mycelia of WT and C-*FgLAI12* produced stearic acid and *cis*-9,*trans*-11 CLA, compared with no *cis*-9,*trans*-11 CLA and a small quantity of stearic acid for the extract from Δ*FgLAI12*. After addition of *cis*-9,*trans*-11 CLA, the enzyme extract from Δ*FgLAI12* produced stearic acid as for the WT. FgLAI12 contains no known conserved domain of transcription factors, further suggesting that FgLAI12 is directly responsible for conversion of LA to cis-9,trans-11 CLA. These results showed that *FgLAI12* encoded a LAI enzyme that catalysed the transformation of LA to *cis*-9,*trans*-11 CLA (Fig. 1), and that *FgLAI12* played a major role in the isomerism of LA in *F. graminearum* under the experimental conditions.

**Figure 2:**
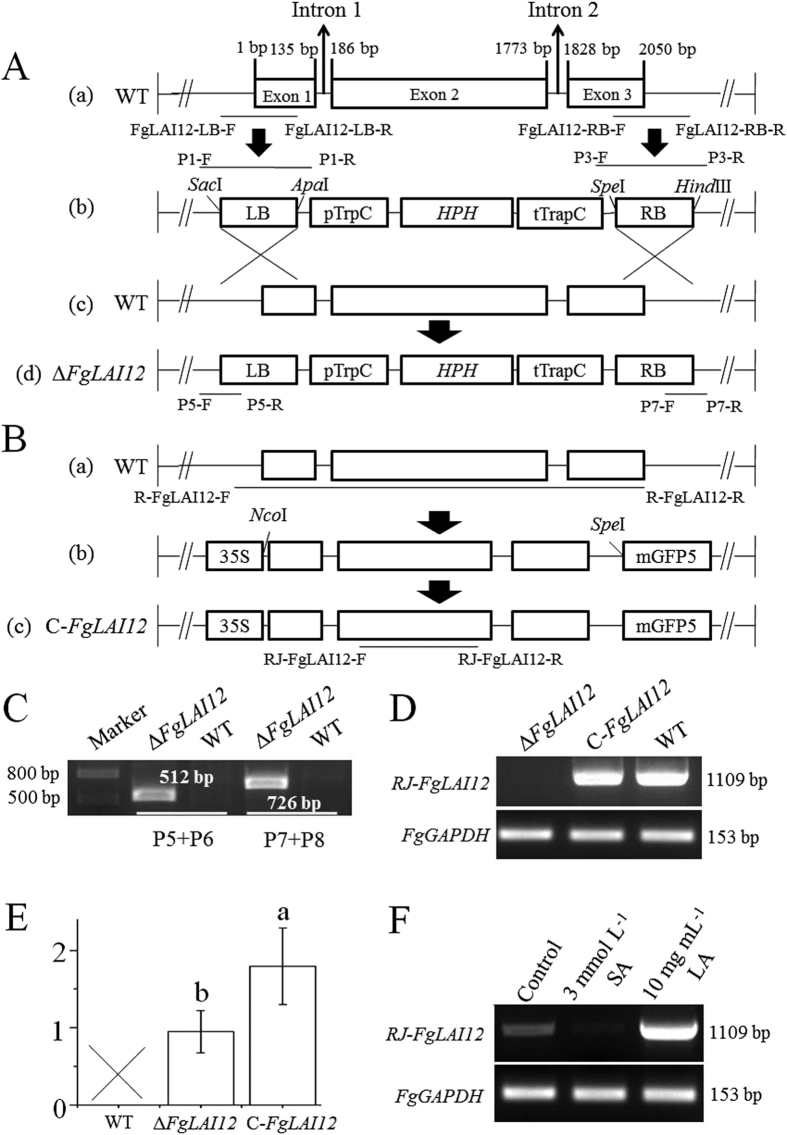
Deletion and complementation of *FgLAI12* in *F. graminearum*. (**A**) The left border (LB) and right border (RB) were amplified from the wild type (a). Δ*FgLAI12* mutant (d) was created by recombination between the knockout vector (b) and *FgLAI12* (c). (**B**) The nucleotide sequence of *FgLAI12* was cloned (a), and ligated into the complementation vector (b). The T-DNA region of the complementation vector was recombined into Δ*FgLAI12* to create C*-FgLAI12* (c). *Sac*I, *Apa*I, *Spe*I, *Hind*III, *Nco*I and *Spe*I indicate the restriction enzymes used. (**C**) PCR verification of Δ*FgLAI12* using the primer pairs P5 forward + P5 reverse and P7 forward + P7 reverse. (**D**) Verification of expression of *FgLAI12* using the primer pair RJ-*FgLAI12* forward + RJ-*FgLAI12* reverse. *FgGAPDH* were used as a control. (**E**) Copy numbers of *HPH* determined by qPCR. Different letters (a and b) above each column indicate a significant difference (*P* < 0.05; n = 3). (**F**) Effect of SA and LA on expression of *FgLAI12* in hyphae, as determined using the primer pair RJ-*FgLAI12* (Table 1). Hyphae were collected on the 4^th^ day after adding SA and LA. *FgGAPDH* was used as a reference. “F”, forward; “R”, reverse.

**Figure 3:**
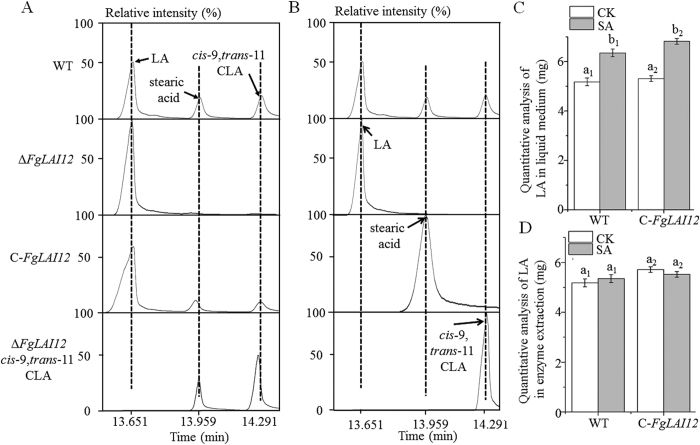
*FgLAI12* encodes a *cis*-12 LA isomerase. (**A**) GC-MS analysis of chemicals derived from LA. (**B**) The peaks for LA, stearic acid and *cis*-9,*trans*-11 CLA when using standard chemicals. (**C**) Quantitative analysis of LA added to liquid medium containing growing mycelia. (**D**) Quantitative analysis of LA added to enzyme extract from mycelia. The same amount of mycelia was used. Different letters above each column indicate a significant difference (*P* < 0.05; n = 3). “a_1,_ b_1_” and “a_2,_ b_2_” are used to show the significance within WT and C-*FgLAI12* treatments, respectively, since there are more than one treatments in the same chart.

SA inhibited the expression of *FgLAI12*^20^ (Fig. 2F). To further confirm the function of *FgLAI12* and clarify the relationship between SA and *FgLAI12*, 3 mL mSNA-2 liquid medium was supplemented with 10 mg SA and cultures of WT, Δ*FgLAI12* and C-*FgLAI12* were incubated for 24 h before collection of mycelia. Compared with cultures that lacked SA, the enzyme extracts from WT and C-*FgLAI12* metabolised a smaller quantity of LA in the presence of SA (Fig. 3C). As expected, following addition of 10 mg SA to the enzyme extract, no significant difference in LA concentration was observed in the presence and absence of SA (Fig. 3D). These results indicated that *FgLAI12* was a target for manipulation of LA metabolism by SA in *F. graminearum*.

### Influence of *FgLAI12* on mycelial growth and conidial morphology

**Figure 4:**
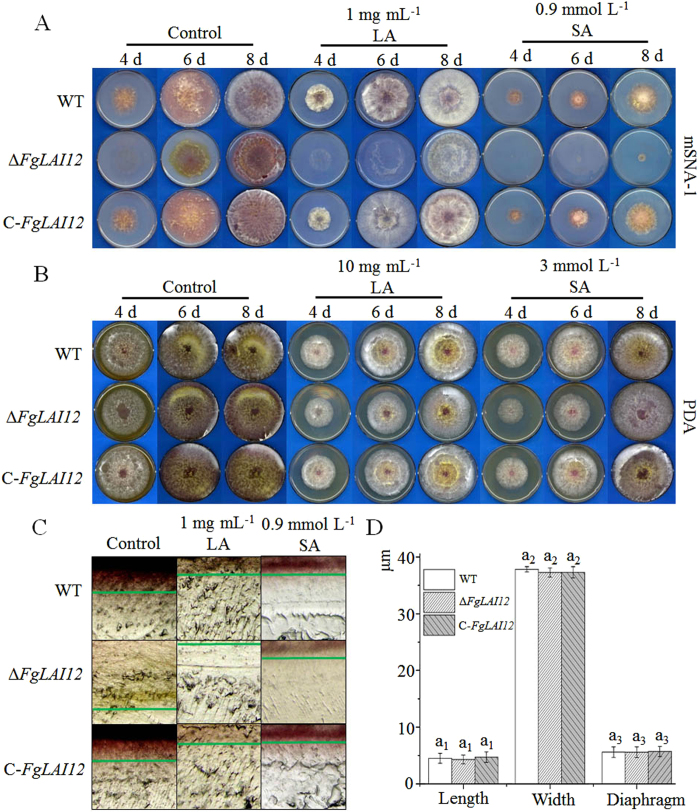
Effect of *FgLAI12* on fungal biology. Mycelial growth of the WT, Δ*FgLAI12* and C-*FgLAI12* on mSNA-1 (**A**) and PDA plates (**B**) under treatment with LA and SA on the 4^th^, 6^th^ and 8^th^ days after inoculation. (**C**) Microscopic observation of mycelia growth within mSNA-1 plates supplemented with LA and SA. The depth of hyphae is marked with green lines. Agar plugs (0.5 cm^2^) cut from the edge of growing mycelia (Fig. 4A) were photographed with an Olympus SZ51 (Japan). (**D**) Comparison of conidial length, width and the number of septa of the WT, Δ*FgLAI12* and C-*FgLAI12*. Different letters above each column indicate a significant difference (*P* < 0.05; n = 1000). The same rule as mentioned in the legend of Fig. 3 is used for indicating the significance within treatment, when there are more than one treatments in the same chart.

To observe the changes in the growth phenotype caused by disruption of *FgLAI12* in *F. graminearum*, the WT, Δ*FgLAI12* and C-*FgLAI12* strains were inoculated on modified Synthetischer Nährstoffarmer Agar-1 (mSNA-1) (Fig. 4A) and potato dextrose agar (PDA) plates (Fig. 4B). On mSNA-1 plates, Δ*FgLAI12* formed much less aerial mycelium compared with WT and C-*FgLAI12*. On the 6^th^ day after inoculation, aerial mycelium of Δ*FgLAI12* was clearly observed (control in Fig. 4A). Similarly, Δ*FgLAI12* mycelia grew more slowly than those of WT and C-*FgLAI12* on PDA plates, as indicated on the 4^th^ day after inoculation (control in Fig. 4B). Compared with the control, the spread of mycelia was inhibited by LA (LA treatment in Fig. 4A,B). Consistent with the role of *FgLAI12* in LA metabolism, the spread and density of Δ*FgLAI12* mycelia were more strongly inhibited by LA. Mycelial growth of Δ*FgLAI12* was more sensitive to SA compared with that of WT and C-*FgLAI12* (SA treatment in Fig. 4A,B). These results indicated that *FgLAI12* plays important roles in mycelial growth and fungal response to SA.

To clarify the influence of *FgLAI12* on fungal growth within agar, a 0.5-cm^2^ agar plug that excluded aerial mycelia was cut from the edge of 6-d-old mSNA-1 plates, and was observed using a stereomicroscope at 100× (Fig. 4C). The hyphae of Δ*FgLAI12* grew more deeply on mSNA-1 plates than hyphae of WT and C-*FgLAI12*. In the presence of LA or SA, the hyphae of WT, Δ*FgLAI12* and C-*FgLAI12* grew more shallowly than those of the controls. Interestingly, the hyphae of Δ*FgLAI12* grew more shallowly under LA treatment than those of WT and C-*FgLAI12.* No notable difference between the strains under SA treatment.

To clarify whether *FgLAI12* affected conidial morphology, we compared the length, width and number of septa of 1 × 10^3^ randomly selected conidia. No significant difference among WT, Δ*FgLAI12* and C-*FgLAI12* was observed for each parameter (Fig. 4D).

### Subcellular localisation of FgLAI12 protein

**Figure 5:**
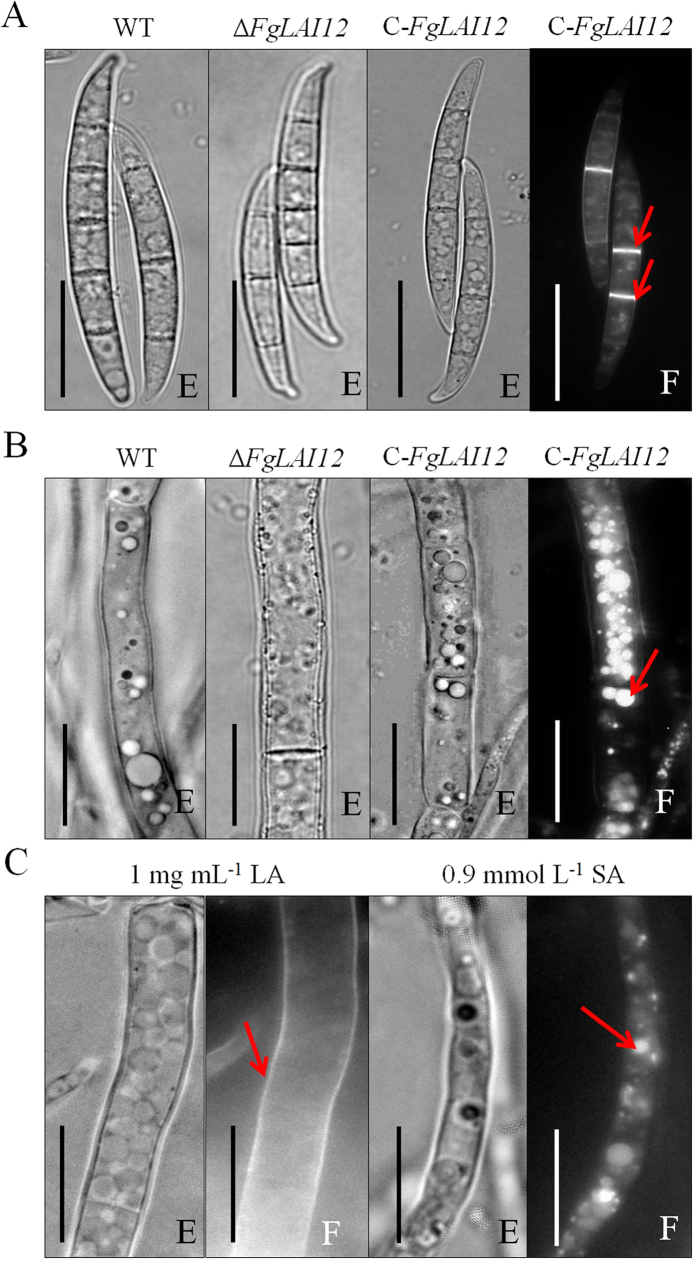
Subcellular localisation of FgLAI12 protein. Conidia (**A**) and mycelia (**B** and **C**) of the WT, Δ*FgLAI12* and C-*FgLAI12* strains photographed with a Nikon-80i fluorescence microscope (Japan). The fluorescent protein signal is marked with red arrows. (**E**), Optical microscope; (**F**), fluorescence microscope. Scale bar, 10 μm.

The C-*FgLAI12* strain harbouring a green fluorescent protein gene (GFP) tag was used to examine the subcellular localisation of FgLAI12 protein. Conidia and hyphae at different vegetative growth stages were used to detect GFP fluorescence. As expected, no morphological differences among conidia of the WT, Δ*FgLAI12* and C-*FgLAI12* strains were observed (Fig. 5A). FgLAI12 protein was predominantly localised on septa of conidia (Fig. 5A), and in vacuoles within hyphae (Fig. 5B). In response to LA treatment, FgLAI12 protein was mainly localised on the cell membrane (Fig. 5C). In the presence of exogenous SA, FgLAI12 protein remained localised in vacuoles but the fluorescence intensity was reduced, reflecting the inhibitory effect of SA on *FgLAI12* expression (Fig. 5C).

### *FgLAI12* affects pathogenicity in wheat

**Figure 6:**
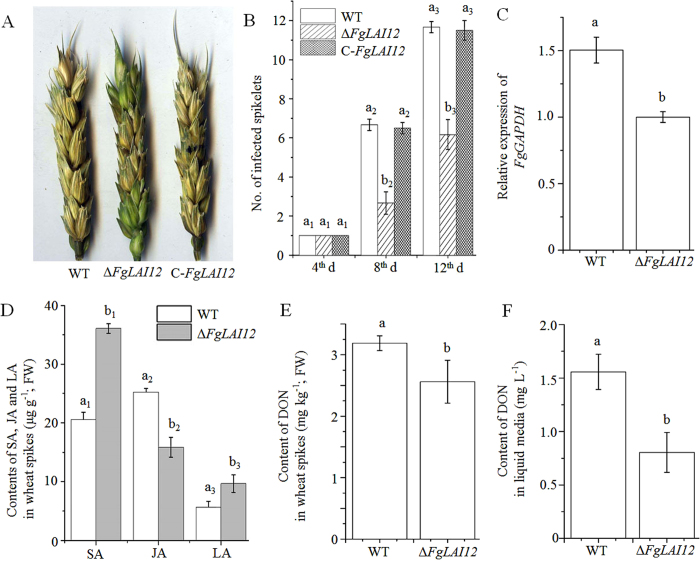
Effect of *FgLAI12* on pathogenicity of *F. graminearum* in wheat. (**A**) Symptoms of Fusarium head blight in spikes of the WT, Δ*FgLAI12* and C-*FgLAI12* strains on the 12^th^ day after inoculation. (**B**) Numbers of infected and bleached spikelets on the 4^th^, 8^th^ and 12^th^ days after inoculation. (**C**) Relative expression level of *FgGAPDH* in wheat spikes. (**D**) Concentrations of salicylic acid (SA), jasmonic acid (JA) and linoleic acid (LA) in spikes inoculated with WT and Δ*FgLAI12*. (**E**) Concentration of deoxynivalenol (DON) in wheat spikes. (**F**) Measurement of DON in liquid medium (the same amount of mycelia was used). Values are the mean ± standard deviation of three biological replicates per treatment. Different letters above each column indicate a significant difference (*P* < 0.05; n = 3). FW, fresh weight. The same rule as mentioned in the legend of Fig. 3 is used for indicating the significance within treatment, when there are more than one treatments in the same chart.

To clarify whether *FgLAI12* was involved in the pathogenicity of *F. graminearum*, we point-inoculated two fully developed florets of a central spikelet within a spike with conidial suspensions of WT, Δ*FgLAI12* and C-*FgLAI12*. Δ*FgLAI12* spikes showed much lower severity of disease compared with those of WT and C-*FgLAI12* (Fig. 6A,B). *FgLAI12* was associated with variation in fungal biomass in wheat spikes, which was indicated by the relative expression level of the *F. graminearum GAPDH* gene. The spikes inoculated with Δ*FgLAI12* conidia showed a significantly lower *GAPDH* expression level than spikes inoculated with WT conidia (Fig. 6C). In addition, the concentration of DON in the liquid culture medium and in wheat spikes was compared. Δ*FgLAI12* showed much lower DON production than that of WT (Fig. 6E,F).

Both SA, jasmonic acid (JA) and LA are important components in the wheat defence response against *F. graminearum*, and infection of spikes by *F. graminearum* leads to a significant increase in their accumulation^6^,^21^. To further understand the reduced disease severity of Δ*FgLAI12* spikes compared with WT spikes, we compared the amount of SA, JA and LA in spikes inoculated with WT and Δ*FgLAI12* conidia. Spikes infected with Δ*FgLAI12* accumulated higher concentrations of SA and LA, and a lower concentration of JA (Fig. 6D).

## Discussion

*Fusarium graminearum* is a fungal pathogen that causes severe damage to wheat crops worldwide. The genetics and biology of *F. graminearum* have been well studied. However, the metabolism of LA and its role in fungal biology and pathogenicity has attracted little attention. The *FgLAI12* gene of *F. graminearum* encodes a *cis*-12 LAI. Our results demonstrated that this *cis*-12 LAI was responsible for transformation of LA to *cis*-9,*trans*-11 CLA. In addition, *FgLAI12* was necessary for fungal growth, fungal response to SA, aerial mycelium development, synthesis of a mycotoxin and pathogenicity in wheat.

Both LDS^8^,^9^ and LAI metabolise LA in fungi (Fig. 1). We previously reported that SA downregulates expression of *FgLAI12*, which encodes a predicted LDS enzyme^20^. Surprisingly, GC-MS analysis demonstrated that FgLAI12 functioned as a LAI *in vivo*. Deletion of *FgLAI12* had no significant effect on conidial production (data not shown) and morphology (Fig. 4D), even though *FgLAI12* played a major role in the isomerism of LA in *F. graminearum* (Fig. 3A). Disruption of LDS expression in *Emericella nidulans* and *Neosartorya fumigata* results in a decrease in ascospore production^8^, because one product of LDS activity, (8 R)-hydroperoxylinoleate, is a sexual sporulation hormone^22^. These results indicate the functional difference of LDS and LAI in fungal biology. Additional studies of *LDS* genes would help to understand the role of LA in *F. graminearum*.

LA plays an important role in wheat and barley in defence against *F. graminearum* and has been established to be a FHB resistance-related metabolite^6^,^7^,^23^. Higher concentrations of LA accumulate in FHB-resistant wheat lines than in susceptible lines^7^, which suggests that LA contributes to FHB resistance. LA is a precursor in the biosynthesis of the phytohormone JA, which is a critical signalling molecule for wheat resistance against FHB^21^,^24^. Spikes inoculated with Δ*FgLAI12* conidia accumulated greater quantities of LA than those inoculated with WT conidia (Fig. 6D), which was consistent with the hypothesised function of *FgLAI12*. Unexpectedly, spikes inoculated with Δ*FgLAI12* accumulated lower concentrations of JA, possibly because of the lower fungal biomass (Fig. 6C). Besides being a chemical inhibitor, LA can reinforce the cuticle, which acts as a barrier to pathogen entry^7^. In this regard, the lower disease severity of Δ*FgLAI12*-inoculated spikes compared with spikes inoculated with WT is understandable.

Fluorescent microscopic observation showed that FgLAI12 protein was usually localised in the septa of conidia and the vacuoles of hyphae. However, exogenous LA treatment resulted in localisation of FgLAI12 in the cell membrane of hyphae, which might reflect the mechanism by which FgLAI12 protein metabolises LA synthesised by the host plant.

SA and JA are important defence phytohormones, for which both antagonistic and synergistic interactions are reported^25^,^26^,^27^,^28^. Both SA and JA accumulate in wheat spikes infected by *F. graminearum*, which indicates that synergistic interactions between the two phytohormones may contribute to FHB resistance^21^. Expression of *FgLAI12* was downregulated by SA^20^, and degradation of LA was reduced in response to exogenous SA (Fig. 3C). Spikes inoculated with Δ*FgLAI12* accumulated higher quantities of SA and reduced amounts of JA compared with spikes inoculated with the WT strain (Fig. 6D), indicating that *FgLAI12* plays a role in manipulating biosynthesis of SA and JA *in planta*. Therefore, we speculate that *FgLAI12* represents a link between the SA and JA defence-response pathways during the host–pathogen interaction, which warrants future investigation.

Mycelial growth of Δ*FgLAI12* was reduced on mSNA-1 and PDA plates compared with that of WT (Fig. 4A). This finding suggests that *FgLAI12* is essential for normal growth of *F. graminearum*. Polyunsaturated fatty acids (PUFAs), such as γ-linolenic, arachidonic and eicosapentaenoic acids, can modify membrane-bound proteins, ATPase and the histocompatibility complex, and control the reaction of fatty acid binding proteins^29^,^30^. As a PUFA, LA may perform a similar function to other PUFAs. Disruption of *FgLAI12* is likely to have disturbed the balance of PUFAs and negatively affected growth of fungal mycelia.

The mycotoxin DON is a threat to human and animal health^1^. Many countries have adopted a maximum allowable level for DON in grains for human consumption and animal feedstuffs. We previously showed that SA significantly reduced DON production^20^ and SA also significantly inhibited expression of *FgLAI12*. In the present study, we demonstrated that disruption of *FgLAI12* reduced accumulation of DON in wheat spikes and in the culture medium. Therefore, *FgLAI12* may be a regulatory target for SA to downregulate DON production in *F. graminearum*.

*FgLAI12* catalyses the transformation of LA to *cis*-9,*trans*-11 CLA. CLA has been shown to lower cancer risk, enhance immunity and reduce body fat while increasing lean body mass in animals^31^,^32^. The *cis*-9,*trans*-11 CLA and *trans*-10,*cis*-12 CLA isomers show the highest biological activities and are transformed by *cis*-12 and *cis*-9 isomerases, respectively (Fig. 1). CLA is currently marketed as a dietary supplement, which is produced by alkaline isomerization of LA^33^ or vegetable oils containing triglyceride esters of LA^34^. However, chemically synthesised CLA contains a variety of other isomers and is unsuitable for nutritional purposes^35^. Industrial-scale production of CLA by biotransformation from LA is increasingly attractive. Therefore, identification of the genes encoding LAI enzymes is warranted. The *cis*-12 *LAI* gene characterised in this study is potentially valuable for commercial use.

## Methods

### Experimental materials and growth conditions

Plants of *Triticum aestivum* ‘Roblin’ were grown in greenhouses under 16/8 h (day/night) cycles at 23/18 °C. Plants were watered as needed and fertilised before planting with 15-15-15 (N-P-K) fertiliser. ‘Roblin’ is highly susceptible to *F. graminearum* infection.

The *F. graminearum* isolate DAOM180378 (Canadian Fungal Culture Collection, AAFC, Ottawa, ON, Canada), which is highly virulent on wheat, was used in all experiments. This isolate was also used for measurement of the expression level of *FGSG_02668 (FgLAI12*) under SA treatment^20^. The isolate was cultured on modified mSNA-1 (1 g KH_2_PO_4_, 1 g KNO_3_, 0.5 g MgSO_4_, 0.5 g KCl, 1 g glucose, 1 g sucrose and 20 g agar per litre) plates at 25 °C. Conidia were produced in carboxymethyl cellulose liquid medium at 28 °C, with shaking (180 rpm) for 5 d^36^. For mycelial growth studies, PDA (BD Difco, Sparks, MD, USA) and mSNA-1 plates were used. LA and SA were added to media after autoclaving. Each plate was inoculated with 1 × 10^3^ conidia of *F. graminearum* and all inoculated plates were incubated in a dark cabinet at 28 °C. There were 10 replicates per treatment and the growth experiments were repeated three times. For deletion and complementation of the *FGSG_02668* gene, *Agrobacterium tumefaciens* strain AGL-1, which was used for transformation of *F. graminearum*, was grown at 28 °C in yeast extract broth (5 g nutrient broth, 1 g yeast extract, 5 g peptone, 5 g sucrose and 0.5 g MgSO_4_ per litre; pH 7.4). *Escherichia coli* strain DH5α (Tiangen, Beijing, China) was cultured in Luria–Bertani broth at 37 °C^37^. Unless specifically noted, all chemicals were purchased from Sigma-Aldrich (St Louis, MO, USA).

### Sequence analysis and primer design

The nucleotide sequence of *FGSG_02668* was downloaded from the Ensembl Fungi database (http://fungi.ensembl.org/index.html). Primer Premier 5.0 software (Premier Biosoft, Palo Alto, Canada) was used to design PCR primers (Table 1).

### Disruption and complementation of *FGSG_02668*

Genomic DNA of *F. graminearum* was extracted from mycelia, which were cultured on mSNA-1 plates for 5 d at 25 °C, by the CTAB method^38^. Disruption of *FGSG_02668* in *F. graminearum* is schematically shown in Fig. 2A. The pRF-HU2 vector was used for targeted gene replacement in *F. graminearum* through *A. tumefaciens*-mediated transformation^39^,^40^. The left border (LB) and right border (RB) of *FGSG_02668* targeted for homologous recombination were amplified with the primer pairs FgLAI12-LB forward + FgLAI12-LB reverse and FgLAI12-RB forward + FgLAI12-RB reverse, respectively. The resulting recombinant plasmids were verified by PCR and sequencing (Invitrogen, Shanghai, China) with the primer pairs P1 forward + P1 reverse and P3 forward + P3 reverse (Fig. 2A). *Agrobacterium*-mediated transformation of *F. graminearum* was performed as described previously^41^. The resulting mutants were verified by PCR and sequencing (Invitrogen) (Fig. 2A,C). The full open reading frame of *FGSG_02668* was inserted into the pCAMBIA1302 vector. The 3′ terminus of *FGSG_02668* was fused with a *GFP* tag under the control of the 35 S promoter. The recombinant pCAMBIA1302 vector was transformed into Δ*FgLAI12* to create complementation mutants. The complementation mutants were verified by PCR and sequencing (Fig. 2B,D). Five knockout mutants and four complementation mutants were used throughout the research.

To confirm the single integration of the *FGSG_02668* knockout construct into the genome, quantitative real-time PCR (qPCR) was performed to determine the copy number of T-DNA inserts in fungal transformants as described previously^42^, with some modification. Genomic DNA was used as the qPCR template. The *HPH* gene was used to determine the copy number of the integrated T-DNA construct for gene knockout. The primer pairs Fg-GAPDH forward + Fg-GAPDH reverse, Fg-β-tubulin forward + Fg-β-tubulin reverse, and Fg-Factor1 forward + Fg-Factor1 forward, which target the single-copy house-keeping gene *FgGAPDH (FGSG_*06257), β-tubulin (*FGSG_09530*) and elongation factor 1 (*FGSG_08811*) of *F. graminearum*, respectively, were used as references for normalisation of data. The primer pair HPH-F + HPH-R was used to amplify the *HPH* gene in qPCR reactions. The qPCR analyses were carried out as described previously^43^ using a MyiQ Real-Time PCR Detection System (Bio-Rad, Hercules, CA, USA).

### RT-PCR analyses

Total RNA was extracted from 100 mg (fresh weight) of mycelia grown on mSNA-1 plates using the E.Z.N.A.^®^ Total RNA Kit I (Omega Bio-Tek, Norcross, GA, USA) in accordance with the manufacturer’s instructions. RNA was reverse transcribed using the PrimeScript™ RT Reagent Kit with genomic DNA Eraser (Takara, Dalian, China) following the protocol of the manufacturer. The primer pair RJ-*FgLAI12* forward + RJ-*FgLAI12* reverse was used to measure the expression level of *FGSG_02668* by RT-PCR. The *FgGAPDH* gene was used as a reference.

### LAI enzyme activity assay

The crude enzyme was extracted as described previously^13^, which was utilised for extacting recombinant LDS enzyme. Mycelia of the WT, Δ*FgLAI12* and C-*FgLAI12* strains were cultured and collected on liquid mSNA-2 (1 g KH_2_PO_4_, 1 g KNO_3_, 0.5 g MgSO_4_, 0.5 g KCl, 6 g glucose and 6 g sucrose per litre) medium, and incubated at 28 °C on an orbital shaker for 6 d in darkness at 120 rpm. The collected mycelia (100 mg fresh weight) were supplemented with 0.1 mol L^−1^ sodium borate and 10 mg LA, then incubated with shaking (90 rpm) for 4 h at 28 °C. Subsequently, mycelia were collected by filtration and dried with filter paper, before being ground in liquid nitrogen. For each strain, three biological replicates were performed.

One millilitre of enzyme extraction solution (0.2 mol L^−1^ sucrose, 0.1 mol L^−1^ Tris, 10 mmol L^−1^ ethylene diamine tetraacetic acid, 0.1 mol L^−1^ sodium borate and 20% [v/v] glycerin; pH 8.0) was added to each 50 mg of mycelia powder. The supernatant was collected by centrifugation at 1 × 10^4^ rpm (Eppendorf centrifuge 5417 R, Hamburg, Germany) for 10 min at 4 °C, and then immediately mixed with 10 mg LA for 1 h at 28 °C. Finally, the targeted chemicals were enriched with SepPak/C_18_, eluted with 0.5 mL methyl alcohol, and dried over Na_2_SO_4_. The enriched samples were analysed by preparative GC-MS. A GC system (6890 N Network, Agilent, China) with a non-polar column (30 m, DB-5; J&W Scientific, Folsom, CA, USA; film, 0.25 μm; diameter, 0.25 mm; carrier gas He, 15 psi; flow rate, 1 mL min^−1^) and MS detector (5973 Network; Agilent, USA) were used. After splitless injections of samples in heptane, the GC was programmed from 80 °C for 4 min, to 200 °C at a rate of 30 °C min^−1^, to 233 °C at 3 °C min^−1^ and then to 285 °C at 10 °C min^−1^. The split ratio was 1:25 and the injected sample size was 1 μL. The standard chemicals for GC-MS (LA, stearic acid and *cis*-9,*trans*-11 CLA) were purchased from Sigma-Aldrich (St Louis, MO, USA).

### Virulence assay

To determine whether *FGSG_02668* affected the severity of FHB disease symptoms in wheat spikes, two florets of a single central spikelet per spike were each point-inoculated with a micropipette at the mid-anthesis stage with 1 × 10^3^ conidial suspension of *F. graminearum*. The spikes were enclosed in plastic wrap, and placed in a room for 48 h at 25 °C under 16/8 h (day/night) cycles. Blight symptoms were assessed on the 4^th^, 8^th^ and 12^th^ days after inoculation. Five to ten plants were used per treatment.

To examine whether disruption of *FGSG_02668* impacted on DON synthesis, the accumulation of DON in liquid media and wheat spikes were tested. Measurement of DON content in liquid media was carried out following a two-stage protocol as described previously^20^,^44^. Two fully deveoped florets of each spikelet of one head were each point inoculated with a micropipette at the mid-anthesis stage with 1 × 10^3^ conidia of *F. graminearum*. The inoculated spikes were treated as above to induce FHB disease. On the 4^th^ day after inocultion, each spikelet was harvested and ground in liquid nitrogen. Three biological replicates were conducted with at least six heads per treatment. Measurement of DON accumulation in wheat spikes was performed as described preciously^45^. The DON concentrations were measured using the DON ELISA kit (Beacon, Hebei, China) and Multiskan Spectrum (Thermo Scientific, Finland).

### Measurement of SA, JA, LA and fungal biomass in wheat spikes

To prepare spike samples, two florets of each fully developed spikelet in an entire spike at mid-anthesis were inoculated with 1 × 10^3^ conidia. After inoculation, the wheat plants were placed in a room as described above. At 48 h after initial inoculation, the spikes were harvested and ground to fine powder in liquid nitrogen for chemical measurement and RNA extraction. Three biological replicates were conducted with at least six heads per treatment. Quantification of SA, JA and LA was performed as described previously^46^.

RNA was extracted as described above. The relative amount of *F. graminearum* was estimated by measuring the expression level of *FgGAPDH* in the RNA samples by qPCR, with normalisation to the three wheat reference genes (w-GAPDH, NCBI UniGene Ta.66461; Aox, Ta.6172; hn-RNP-Q, Ta.10105)^20^.

### Microscopic assay

For optical microscopic and fluorescence microscopic assays, conidia were produced in carboxymethyl cellulose liquid medium at 28 °C with shaking (180 rpm) for 5 d. Conidia (1 × 10^3^) were inoculated into 3 mL mSNA-2 liquid media, incubated at 28 °C on an orbital shaker for 6 d in darkness at 120 rpm, and then 20 mg LA was added 1 d before microscopic observation. The conidia and hyphae were observed with a Nikon-80i fluorescence microscope (Nikon, Japan).

### Statistical analysis

Student’s *t*-test (implemented in DPS version 12.01 software) was used to test the significance of differences in the means for LA metabolism, conidial morphology, DON production and disease severity between treatments.

## Additional Information

**How to cite this article:** Zhang, Y.-Z. *et al*. Linoleic acid isomerase gene *FgLAI12* affects sensitivity to salicylic acid, mycelial growth and virulence of *Fusarium graminearum. Sci. Rep.* **7**, 46129; doi: 10.1038/srep46129 (2017).

**Publisher's note:** Springer Nature remains neutral with regard to jurisdictional claims in published maps and institutional affiliations.

## Figures and Tables

**Table 1:** Primers used in this study.

Primer	Forward (5′–3′)	Reverse (5′–3′)
*FgLAI12*-LB	GGAAGCTTCAAAGCGGCAACAC	GGACTAGTTTCCCATCAACAACATAT
*FgLAI12*-RB	GCGGGCCCGGCTTATCTCAACGACA	GCGAGCTCTACCCTGAATCCAACAT
P1^45^	CTTTTCTCTTAGGTTTACCCG	TAATGCAGGAGTCGCATAAG
P3^45^	CCCAAAAAGTGCTCCTTCAA	TGTGCTGCAAGGCGATTAA
P5^45^	TTGTCACCGCCAAAGC	ACCTACTACTGGGCTGCTT
P7^45^	CTGACCAGTTGCCTAA	ACAGTTCATTCCGAGAC
R-*FgLAI12*	GGACTAGTTGCTCAACTTACGCCACTG	GGCACGTGTCGACGCAGAAGCT
RJ-*FgLAI12*	ATCCCAAGCCACCAG	GCCAAGTTCTCCGTCA
Fg-GAPDH^20^	TGACTTGACTGTTCGCCTCGAGAA	ATGGAGGAGTTGGTGTTGCCGTTA
w-GAPDH^20^	AACTGTTCATGCCATCACTGCCAC	AGGACATACCAGTGAGCTTGCCAT
Aox^20^	GACTTGTCATGGTAGATGCCTG	CAGGACGAGCATAACCATTCTC
hn-RNP-Q^20^	TCACCTTCGCCAAGCTCAGAACTA	AGTTGAACTTGCCCGAAACATGCC
Fg-β-tubulin^20^	GTTGATCTCCAAGATCCGTG	CATGCAAATGTCGTAGAGGG
Fg-Factor1^20^	CCTCCAGGATGTCTACAAGA	CTCAACGGACTTGACTTCAG
*HPH*	CGATCTTAGCCAGACGAGCG	TTGCCCTCGGACGAGTGCTG

Horizontal lines under nucleotides indicate the cutting sites for restriction enzymes.
